# Role of myeloid regulatory cells (MRCs) in maintaining tissue homeostasis and promoting tolerance in autoimmunity, inflammatory disease and transplantation

**DOI:** 10.1007/s00262-018-2264-3

**Published:** 2018-10-24

**Authors:** Giada Amodio, Joanna Cichy, Patricia Conde, Gianluca Matteoli, Aurélie Moreau, Jordi Ochando, Barbaros H. Oral, Michaela Pekarova, Elizabeth J. Ryan, Johannes Roth, Yahya Sohrabi, Maria-Cristina Cuturi, Silvia Gregori

**Affiliations:** 1 San Raffaele Telethon Institute for Gene Therapy (SR-Tiget), San Raffaele Scientific Institute IRCCS, Via Olgettina, 58, 20132 Milan, Italy; 2 Faculty of Biochemistry, Biophysics and Biotechnology, Jagiellonian University, Krakow, Poland; 3 Centro Nacional de Microbiologia, Instituto de Salud Carlos III, Majadahonda, 28220 , Madrid, Spain; 4 Translational Research in Gastrointestinal Disorders (TARGID), Department of Chronic Diseases, Metabolism and Ageing, KU Leuven, Leuven, Belgium; 5 Centre de Recherche en Transplantation et Immunologie UMR1064, INSERM, Université de Nantes, Nantes, France; 6 Institut de Transplantation Urologie Nephrologie (ITUN), CHU Nantes, Nantes, France; 7 Department of Immunology, Faculty of Medicine, Uludag University, Bursa, Turkey; 8 Institute of Biophysics, The Czech Academy of Sciences, Brno, Czech Republic; 9 Department of Biological Sciences, Faculty of Science and Engineering, University of Limerick, Limerick, Ireland; 10 Institute of Immunology, University of Münster, Münster, Germany; 11 Molecular and Translational Cardiology, Department of Cardiovascular Medicine, University Hospital Münster, Münster, Germany

**Keywords:** Myeloid regulatory cells (MRCs), Polymorphonuclear neutrophils, Monocytes/macrophages, Dendritic cells, Tolerance, Mye-EUNITER

## Abstract

Myeloid cells play a pivotal role in regulating innate and adaptive immune responses. In inflammation, autoimmunity, and after transplantation, myeloid cells have contrasting roles: on the one hand they initiate the immune response, promoting activation and expansion of effector T-cells, and on the other, they counter-regulate inflammation, maintain tissue homeostasis, and promote tolerance. The latter activities are mediated by several myeloid cells including polymorphonuclear neutrophils, macrophages, myeloid-derived suppressor cells, and dendritic cells. Since these cells have been associated with immune suppression and tolerance, they will be further referred to as myeloid regulatory cells (MRCs). In recent years, MRCs have emerged as a therapeutic target or have been regarded as a potential cellular therapeutic product for tolerance induction. However, several open questions must be addressed to enable the therapeutic application of MRCs including: how do they function at the site of inflammation, how to best target these cells to modulate their activities, and how to isolate or to generate pure populations for adoptive cell therapies. In this review, we will give an overview of the current knowledge on MRCs in inflammation, autoimmunity, and transplantation. We will discuss current strategies to target MRCs and to exploit their tolerogenic potential as a cell-based therapy.

## Introduction

Dysregulation of the immune system and uncontrolled inflammation contribute to disease pathology. Myeloid cells play a key role in this process: they initiate effective and controlled immune responses that protect the host. However, under certain circumstances, they contribute to the inflammatory process, exacerbating disease pathology. Alternatively, myeloid cells with regulatory properties can protect the host from uncontrolled inflammation that might be triggered by pathogens or self-antigens (Ags). These cells, referred to as myeloid regulatory cells (MRCs), have been described within all the major myeloid cell lineages: polymorphonuclear neutrophils (PMN), macrophages, and dendritic cells (DCs). Moreover, a particular subset of MRCs, termed myeloid-derived suppressor cells (MDSCs) according to their regulatory activity, has been described. MRCs promote a tolerogenic microenvironment that sustains the generation of T-regulatory cells (Tregs), thereby, the induction of tolerance. The ability of MRCs to control immune responses and to promote tolerance has prompted an interest in exploiting them therapeutically to treat inflammation, autoimmunity, or to improve outcomes in transplantation. Here, we present an overview of the role of different MRCs in inflammation, autoimmunity (see “Myeloid regulatory cells in inflammation and autoimmunity”), and in organ transplantation (see “Myeloid regulatory cells in allo-reactive T-cell responses”). We include data from experimental disease models and patients, if available.

## Myeloid regulatory cells in inflammation and autoimmunity

The inflammatory response is a self-limiting process, culminating in the complete resolution of inflammation and a rapid return to tissue homeostasis. Disruption of the tightly regulated mechanisms that control the resolution of inflammation can result in excessive and persistent immune activation, which may cause tissue damage and promote the onset of autoimmune disease. Originally, the resolution of inflammation was considered a passive process. However, strong evidence is emerging that the resolution of inflammation is an active process crucial for preventing uncontrolled inflammation and collateral damage. Myeloid cells, including MRCs, are a key component of the regulatory response and it is imperative to understand the mechanisms underpinning their recruitment and activation. While the suppression inhibition of inflammation in a myeloid cell-dependent manner can be detrimental in cancer and chronic infections (covered/reviewed by Umansky et al. and Dorhoi et al. in companion reviews in this “symposium-in-writing”), it plays a key role in modulating T-cell responses and promoting/maintaining tolerance.

In the following sections, we will discuss the role that different MRCs play in modulating inflammation and autoimmunity both in experimental models and patients. We also review therapeutic approaches targeting MRCs or exploiting MRC-based cell therapy to restore tolerance.

### Contribution of polymorphonuclear neutrophils to inflammation in autoimmunity

Neutrophils are the most abundant circulating leukocytes in humans and the first line of defense against pathogens. They are present in large numbers at sites of autoimmune damage, such as the Rheumatoid Arthritis (RA) synovium, psoriatic skin, or Systemic Lupus Erythematosus (SLE) affected sites, where they contribute to pathology [1]. A reduced frequency of neutrophils in experimental models can lead to different outcomes: in Type 1 diabetes (T1D), this attenuates disease development [2], whereas in Genista mice, it is associated with spontaneous lupus-like autoimmunity [3]. Lower levels of neutrophils are typically associated with reduced disease severity, suggesting that neutrophils participate in promoting inflammation and autoimmunity.

Autoimmune disorders often involve organs that are densely colonized by microbes and frequently exposed to pathogens, e.g., the gastrointestinal tract or skin. Neutrophils are recruited to these sites to fight infection, frequently being the first cells recruited, where they act as effector cells via phagocytosis of the pathogens, release of lytic enzymes, and production of reactive oxygen species and inflammatory mediators [1]. Neutrophils mediate tissue damage by exposing autoAgs (e.g., in autoimmune vasculitis where neutrophils become the target of myeloperoxidase or proteinase three specific autoantibodies), or releasing autoAgs, primarily when dying by apoptosis or through the formation of neutrophil extracellular traps (NETs) [4]. During inflammatory responses, neutrophils interact with natural killer (NK) cells, macrophages, plasmacytoid (p)DCs, T- and B-lymphocytes, or can home to secondary lymphoid organs, where they serve as antigen-presenting cells (APCs) [5], activate autoreactive T-cells [5], and promote B-cell differentiation [6]. In autoimmunity, the best characterized neutrophil cellular partners are pDCs, the main producers of IFN-α and inducers of Th17-mediated inflammation [7]. IFN-α production by pDCs requires the formation of nucleic acid complexes with specific peptides/proteins (e.g., anti-microbial peptide LL37, or neutrophil elastase together with secretory leukocyte protease inhibitor), which activate intracellular Toll-like receptors (TLRs). NETs and NET-like structures containing neutrophil DNA, peptides, and proteins, directly activate pDCs to produce IFN-α [8]. Several lines of evidence indicate that the neutrophil/pDC axis is active in autoimmunity: in psoriatic patients, pDCs are in close proximity to neutrophils and NETs [9]; in SLE patients, neutrophils by extruding oxidized DNA within NETs stimulate pDCs to produce IFN-α [10]; in experimental models of T1D, neutrophils and pDCs accumulate within the pancreas, where they contribute to tissue inflammation and autoantibody production [2].

The abnormalities in neutrophil phenotype and function reported in autoimmune diseases indicate that these cells play an important role in promoting/maintaining aberrant immune responses and tissue damage. However, recent evidence indicates that neutrophils with regulatory activity also exist and can act to suppress T-cell responses [11], opening up the possibility that regulatory neutrophils are involved in dampening/controlling inflammatory responses in autoimmunity. Neutrophils display phenotypic and functional heterogeneity, exemplified in humans by their sub-classification into low-density and “conventional” polymorphonuclear neutrophils (PMNs) [11]. In autoimmune disease, low-density neutrophils promote inflammation. However, under certain conditions, e.g., in cancer, low-density granulocytes are a major constituent of the immunosuppressive cell subset, termed PMN-MDSCs. However, some PMN-MDSCs can pass through the gradient and contaminate the high-density fraction of cells that is generally enriched in conventional PMNs [12]. Thereby, to clarify the role neutrophils play in autoimmune inflammation or tissue homeostasis, a more complete characterization of neutrophil diversity and plasticity is needed. New tools currently under development to dissect neutrophil phenotype and function in vivo will address these questions in the near future (covered/reviewed by Cassetta et al. and [13] in companion reviews in this “symposium-in-writing”).

### Role of monocytes/macrophages to promote/control inflammation in autoimmunity

Circulating monocytes and tissue-resident macrophages are key cells of the innate immune system involved in the pathogenesis of inflammatory and autoimmune diseases. Monocytes and macrophages display a variety of effector functions depending on the activation of specific signaling pathways and on their metabolic adaptation.

Monocytes are highly plastic and heterogeneous, and can be classified into distinct subsets, based on phenotype and function. Human monocytes are classified as follows: CD14^high^CD16^−^ ‘classical’ inflammatory monocytes, the prevalent predominant subset of blood monocytes, CD14^+^CD16^+^ ‘intermediate’ monocytes, and CD14^low^CD16^+^ ‘non-classical’ monocytes [14]. While all monocyte subsets have phagocytic potential and secrete pro-inflammatory cytokines, the ‘intermediate’ CD14^+^CD16^+^ cells and ‘non-classical’ CD14^low^CD16^+^ cells display distinct gene expression profiles from ‘classical’ CD14^high^CD16^−^ monocytes. The majority of IL-10-producing cells are CD14^+^CD16^+^ ‘intermediate’ monocytes and these cells are selectively expanded in different pathologies. In contrast, CD14^low^CD16^+^ ‘non-classical’ monocytes have a reduced phagocytic capacity, produce low amounts of reactive oxygen species, and have the unique ability to patrol the endothelium for signs of damage and infection [14]. The classification of human monocytes resembles that proposed in mice, with ‘classical’ monocytes being Ly6C^high^CX_3_CR1^low^CCR2^high^CD43^low^, ‘intermediate’ monocytes being Ly6C^high^CD43^high^, and ‘non-classical’ monocytes defined as Ly6C^low^CX_3_CR1^high^CCR2^low^CD43^high^ [14].

In recent years, the belief that adult tissue-resident macrophages are replenished by monocytes from the bone marrow has been revised. New evidence has emerged indicating that these immune cells have an embryonic origin and are self-maintaining regardless of bone marrow contribution. This new paradigm increases the complexity of tissue macrophages, indicating that in addition to the phenotypic and functional heterogeneity, populations of macrophages with different ontology co-exist at steady state and during inflammation within tissue [15]. Despite their origin, tissue-resident macrophages have been categorized into classically activated, or pro-inflammatory (M1 and murine Ly6C^high^) and alternatively activated, or anti-inflammatory (M2 and murine Ly6C^low^) macrophages. M1 and murine Ly6C^high^ macrophages are linked to inflammation and autoimmune development, whereas M2 and murine Ly6C^low^ macrophages are associated with fibrosis, allergies, and tumor progression (covered/reviewed by Umansky et al. in companion reviews in this “symposium-in-writing”).

**Fig. 1:**
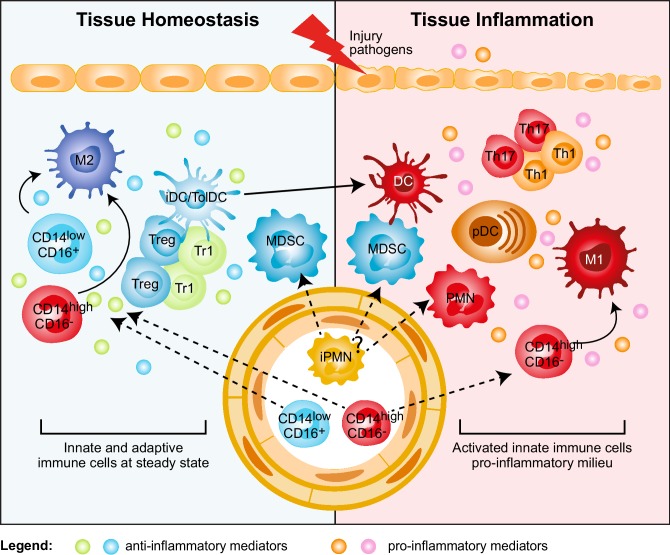
Myeloid regulatory cell contribution in tissue homeostasis and inflammation. Several subsets of myeloid regulatory cells (MRCs) are involved in preventing uncontrolled responses, in maintaining tissue homeostasis, and in promoting resolution of inflammation. Tissue homeostasis. Tissue-resident non-inflammatory M2 macrophages, immature and specialized DC subsets (iDC/TolDC), and MDSCs promote tissue homeostasis via different mechanisms: (1) secretion of anti-inflammatory mediators, such as IL-10 and TGF-β, and expression of IDO; (2) induction of T-regulatory cells, both FOXP3^+^ Tregs and Tr1 cells; (3) generation of a non-inflammatory milieu that leads to the differentiation of migrating classical inflammatory CD14^high^CD16^−^ and not classical CD14^low^CD16^+^ monocytes into anti-inflammatory M2 macrophages, which contribute to T-regulatory cell induction. Tissue inflammation. Upon tissue injury or pathogen entry, PMNs are recruited at the site of inflammation and, by secreting pro-inflammatory mediators, lead to the activation of plasmacytoid DC (pDCs), which consequently release IFN-α. The inflammatory milieu promotes the recruitment of classical inflammatory CD14^high^CD16^−^ monocytes to the site of inflammation and their differentiation into pro-inflammatory M1 macrophages, and the activation and maturation of DCs. These cells in turn promote Th1 and Th17 cell responses via secretion of pro-inflammatory cytokines, such as IL-12 and IL-23. It still remains to be clarified whether MDSCs contribute to tissue inflammation

The selective expansion of peripheral blood CD14^+^CD16^+^ monocytes correlates with disease severity in RA, Inflammatory Bowel Disease (IBD), and psoriasis [16]. A specific reduction of circulating CD14^+^CD16^−^ monocytes in favor of CD14^low^CD16^+^ monocytes has been observed in RA patients responding to therapy [17]. Conversely, activated monocytes are expanded in the synovial fluid of RA patients [18], in the inflamed mucosa of IBD patients [19], and in the central nervous system (CNS) of relapsing remitting Multiple Sclerosis (MS) patients [20]. The massive infiltration of activated CD14^high^CD16^−^ monocytes is a major source of cytokines that disrupts tissue homeostasis by promoting conversion of resident M2 into M1 macrophages (Fig. 1). The role of resident M2 macrophages in maintaining tissue integrity and limiting/resolving inflammation is supported by several lines of evidence both in non-inflamed tissues and experimental models, in which M2 and murine Ly6C^low^ macrophage depletion results in worsened disease [21]. Conversely, experimental models of intestinal inflammation and autoimmune encephalomyelitis (EAE) showed that increased frequency of inflammatory Ly6C^high^ macrophages promotes and sustains tissue damage and aggravates disease symptoms [19, 22]. These examples underline that the balance between M1/M2 macrophages is important for controlling/resolving inflammation. In IBD patients responding to anti-TNFα therapy accumulation of anti-inflammatory M2 macrophages has indeed been associated with mucosal healing [23].

Overall, accumulation and/or persistence of inflammatory monocytes/macrophages within the target organ leads to excessive inflammation and induction of pathogenic cells in autoimmunity. Moreover, the concomitant reduction/impairment of macrophages with immunomodulatory activity sustains inflammation and contributes to disease progression. From a therapeutic point of view, this observation implies that to suppress inflammation and restore tissue homeostasis, the accumulation of anti-inflammatory monocytes/macrophages in the target organ is critical. Moreover, strategies aimed at targeting factors driving the selective differentiation of migrating monocytes into M2 macrophages or preventing the conversion of tissue-resident M2 into M1 macrophages, as already reported in transplantation settings (see below), may be more effective than blocking the development of inflammatory cells.

### Impact of myeloid-derived suppressor cells in autoimmunity

MDSCs are a heterogeneous population of myeloid cells with different maturation stages, and the capacity to suppress immune responses [24]. MDSCs accumulate in the blood, bone marrow, and secondary lymphoid organs of tumor-bearing mice and cancer patients, in whom circulating levels of MDSCs correlate with clinical stage and metastatic burden [24]. In mice, MDSCs are broadly defined as CD11b^+^Gr-1^+^ cells, although they comprise subsets known as granulocytic (PMN)-MDSC (CD11b^+^Ly6G^+^Ly6C^low^) and monocytic (M)-MDSC (CD11b^+^Ly6G^−^Ly6C^high^) cells. Similar to their murine counterpart, human MDSCs comprise two cell subtypes with either granulocyte or monocyte morphology. Human PMN-MDSCs (CD11b^+^CD14^−^CD15^+^ or CD66b^+^) and M-MDSCs (CD11b^+^CD14^+^HLA-DR^−^CD15^−^) are phenotypically overlapping with neutrophils and monocytes, respectively. However, these cells are defined as MDSCs as they display immunosuppressive functions [25]. The lack of consensus on specific markers, which would allow precise MDSC identification, and their phenotypic heterogeneity have generated controversial results regarding the role of MDSC in autoimmune diseases.

In experimental models of autoimmunity, accumulation of PMN-MDSCs in lymphoid and target organs is associated with inhibition of T-cell proliferation and reduction of pro-inflammatory cytokine release [26, 27]. Expansion of PMN-MDSCs has been described in the synovial fluid of RA patients, where they contribute to limiting the expansion of autoreactive T-cells [28], and in the peripheral blood of MS patients with active disease. Furthermore, they suppress the activation and ex vivo proliferation of autologous CD4^+^ T-cells [27]. Moreover, the proportion of MDSCs, comprising both PMN-MDSC and M-MDSC, correlated with disease course in EAE: both MDSC subsets decrease significantly during the remitting phase, and MDSCs completely disappear during the chronic phase [29]. Overall, these examples indicate that MDSCs play an important role in limiting inflammation. However, MDSCs have also been associated with increased inflammatory responses in autoimmunity. Indeed, an accumulation of MDSCs with the ability to promote an inflammatory microenvironment and pathogenic Th17 cells has been described in target tissues in experimental models of RA, EAE, and SLE [24]. Moreover, an increased frequency of circulating M-MDSCs in RA patients and of circulating PMN-MDSCs and M-MDSCs in SLE patients has been correlated with Th17 responses and disease severity [24].

These discrepancies may be explained by the different strategies and markers used to identify MDSCs. Only recently, suggestions on the standardization of gating strategies and markers to be used to distinguish PMN-MDSCs and M-MDSCs have been proposed [25]. Importantly, one of the key characteristics allowing the classification of both PMN-MDSCs and M-MDSCs is their suppressive activity [24, 25]. However, the lack of consensus on the suppressive assays to be used to assess MDSC regulatory activity, as discussed in [13], has limited their definitive classification to date. Thus, to draw conclusions regarding MDSC contribution in the suppression or induction of autoreactive immune responses, consensus on biomarkers to distinguish MDSCs from other myeloid cell types and to discriminate the different MDSC subsets, and standardized methods to define their suppressive properties are warranted.

### Role of dendritic cells in promoting/regulating autoreactive T-cell responses

DCs are professional APCs specialized in the uptake, processing, and presentation of Ags to T-cells. Conventional, e.g. immunogenic DCs, are involved in the initiation of adaptive immune responses. However, in steady state, these conventional DCs or specialized subsets of DCs, termed tolerogenic (tol)DCs, control tissue homeostasis and induce/maintain tolerance [30]. Aberrant activation of immunogenic DCs or defects in the function of tolDCs are involved in breaking self-tolerance in autoimmune disease [30].

Accumulation and activation of conventional DCs in target organs promote autoreactive T-cell activation, and contribute to local inflammation in autoimmunity [30]. Increased numbers of activated conventional DCs with the ability to stimulate autoreactive T-cells and to secrete pro-inflammatory cytokines are evident in synovial fluid of RA patients, in the demyelinating regions of the CNS, in psoriatic skin lesions, and in intestinal mucosa in IBD patients. These cells contribute to effector Th1 and Th17 cell activation and disease progression [30].

Tissue-resident conventional DCs, characterized by the expression of specific markers, such as langerin (CD207) in the skin (Langerhans cells) or CD103^+^ DCs in the intestinal mucosa, perform tolerogenic functions and maintain tissue homeostasis [30]. An additional subset of tolerogenic DCs, are DC-10, characterized by the expression of HLA-G and Ig-like transcript-4 (ILT4) and the ability to promote IL-10-mediated tolerance [31]. These cells are present in secondary lymphoid organs [32] and in human decidua during pregnancy, where they participate in maintaining fetal–maternal tolerance [33].

The regulatory activity of DCs depends both on their immature state and expression of immune-modulatory factors [e.g., IL-10, TGF-β, indoleamine 2,3-dioxygenase (IDO), aryl-hydrocarbon receptor]. These features are controlled and induced by several environmental signals and crosstalk with local immune cells that, as described above, are dysregulated in inflamed tissues (Fig. 1). Therefore, we can speculate that in autoimmunity a pro-inflammatory environment in the target organ, enriched in immune effector cells, leads to an increased number of inflammatory DCs and a reduced frequency of tolDCs or to a breakdown in tolDC regulatory activities. TolDCs and immunogenic DCs express many overlapping cell-surface markers. Therefore, only functional analysis, e.g., cytokine profile, stimulatory or suppressive activity, can be used to fully define them. The identification of specific biomarkers and consensus on the assays to determine tolDC suppressive activity are critical to better define their role in different autoimmune diseases. This knowledge is required to enable the development of targeted interventions to promote tolDC differentiation, recruitment to sites of inflammation, and maintenance of their regulatory function.

### Therapeutic intervention to restore tolerance in autoimmunity

Current therapies to treat autoimmunity are based on systemic administration of immunosuppressive drugs. While often leading to the amelioration of symptoms, these drugs can have widespread side effects and, in many cases, do not promote durable disease remission. Alternatively, biological therapies consisting of antibodies targeting pro-inflammatory cytokines and their receptors can dampen inflammation and may prevent myeloid cell hyperactivation. While efficacious, these treatments are expensive and long-term administration can result in loss of response and cumulative side effects. An innovative and challenging approach to control auto-reactive T-cells and restore tolerance in autoimmunity is to boost the regulatory arm of the immune system by suppling ex vivo generated MRCs (i.e., MDSCs or tolDCs).

Adoptive transfer of ex vivo isolated or in vitro induced M-MDSCs or PMN-MDSCs in experimental models of RA and EAE ameliorated disease severity by reducing Th1 and Th17 immune responses [24]. Conversely, the therapeutic potential of MDSCs in T1D remains an open question: while adoptive transfer of MDSCs cells improved glucose tolerance and insulin resistance [34], in vitro bone marrow (BM)-derived MDSCs cells failed to prevent autoimmunity in vivo [35]. Ag specificity is likely one of the factors contributing to these discrepancies, since infusion of MDSCs conferred protection only in the presence of cognate Ag [36]. The translation of effective MDSC-based therapies into clinical application faces several hurdles: how in vitro induced MDSCs respond to different inflammatory mediators; whether inflammatory mediators may inhibit MDSCs activity in vivo; and, importantly, whether in vitro induced MDSCs can mature and differentiate into conventional DCs and M1 macrophages, thus acquiring the ability to present autoAgs and exacerbate disease.

Human tolDCs potentially suitable for cell-based therapies can be differentiated in vitro using a plethora of agents [37]. The first clinical trial, performed in T1D patients, demonstrated the safety of this tolDC-based cell therapy approach but with limited effects on the patients’ insulin requirements [38]. Monocyte-derived DCs cultured with a nuclear factor-kB (NF-kB) inhibitor, pulsed with citrullinated peptide Ags, and injected in RA patients significantly reduced levels of activated effector T-cells and pro-inflammatory cytokines [39]. DCs differentiated in the presence of vitamin D3 and dexamethasone (VitD3/Dexa) injected into the knee joints of RA patients can stabilize disease symptoms [40]. VitD3/Dexa DCs were also safely intraperitoneally administered to Crohn’s disease patients with active disease, but no clear clinical benefit was observed [41]. A similar approach is currently being used to treat MS patients with active disease: VitD3 DCs will be administered in MS patients via intradermal injection close to cervical lymph nodes (ClinicalTrials.gov identifier: NCT02618902) or directly injected in cervical lymph nodes (TOLERVIT-MS, ClinicalTrials.gov identifier: NCT02903537).

These completed and ongoing studies have demonstrated the safety of the cell-based approach and some clinical benefit. However, several questions remain before tolDC-based cell therapies can be routinely used to treat or cure autoimmune disease, including the route of administration, the maintenance of the tolerogenic cell properties in vivo, and the ability to stably present autoAgs to inhibit auto-reactive T-cells while promoting autoAg-specific Tregs, thus re-establishing long-standing tolerance.

## Myeloid regulatory cells in allo-reactive T-cell responses

**Fig. 2:**
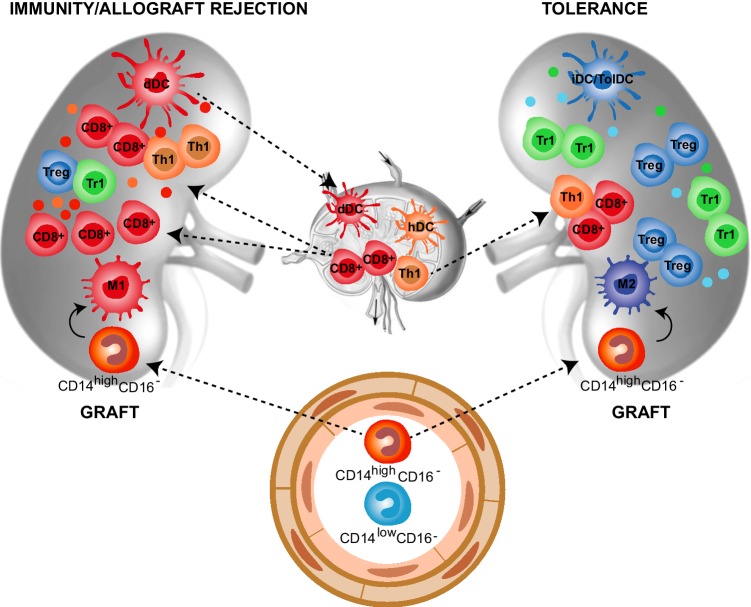
Myeloid cells in allograft rejection and tolerance. Myeloid cells play a central role in allograft rejection and tolerance induction after transplantation. Immunity/allograft rejection. Donor myeloid DCs (dDCs) migrate to the secondary lymphoid organs and activate recipient allo-reactive effector CD8^+^ and CD4^+^ (Th1) cells, which migrate back into the graft where they mediate rejection. Moreover, dying dDCs in the draining lymph nodes release alloAgs, host DCs (hDCs) uptake donor-derived alloAgs and contribute to the activation of alloAgs-specific effector CD8^+^ and CD4^+^ (Th1) cells. Within the graft, classical inflammatory CD14^high^CD16^−^ monocytes are recruited from the circulation and differentiate into M1 macrophages that, by secreting pro-inflammatory mediators, contribute to the expansion of effector alloAg-specific T-cells. The limited number of T-regulatory cells (Tregs and Tr1 cells) present within the graft is not sufficient to control the massive infiltration of effector alloAg-specific T-cells. Tolerance. The graft microenvironment enriched of anti-inflammatory mediators, including IL-10, TGF-β, and CSF-1, leads to the differentiation of migrating classical inflammatory CD14^high^CD16^−^ monocytes into anti-inflammatory M2 macrophages, which promote alloAgs-specific T-regulatory (Treg and Tr1) cells. In addition, the recruitment and/or induction of immature and tolerogenic DC subsets (iDC/TolDC) within the graft sustains the expansion/induction of alloAgs-specific T-regulatory (Treg and Tr1) cells, leading to long-term transplantation tolerance

Organ transplantation is the most efficient treatment to replace the loss of organ function in patients suffering from end-stage diseases. Graft rejection remains a major limitation of organ transplantation. Myeloid cells are involved both in innate non-specific reactions and donor-specific adaptive responses during allograft rejection. Three pathways promote allo-specific T-cell activation after organ transplantation [42]. In the direct pathway, after transplantation, donor APCs, mainly DCs, migrate from the graft into recipient secondary lymphoid organs, where they present alloAgs to host naive T-cells. These activated T-cells differentiate into effector T-cells, that migrate back to the graft, where they can mediate rejection (Fig. 2). In the indirect pathway, host T-cells are primed in the secondary lymphoid organs by host APCs that uptake alloAg derived from dying migrated donor DCs (Fig. 2) [43]. Finally, in the semi-direct pathway host APCs acquire intact allogeneic MHC-peptide complex from donor APCs by direct cell-to-cell contact or via exosomes, leading to host T-cell stimulation [44]. The direct and the indirect pathway are mainly involved in early acute and chronic graft rejection, respectively.

Current pharmacological approaches to prevent graft rejection rely on long-term and non-specific therapies that result in metabolic toxicity and other undesirable side effects, such as infection and cancer. Consequently, graft survival outcomes are suboptimal. Novel therapeutic approaches that target adaptive immune responses are currently under clinical testing. These approaches include the use of costimulatory blockade, lymphodepletion, or in vivo induction of Tregs. While promising results have been obtained, transplantation tolerance, defined as a state of donor-specific unresponsiveness, remains elusive [45]. This underlines the need of developing alternative tolerance-inducing protocols. In the present section, we discuss the role of different subtypes of MRCs in modulating allograft rejection in experimental models and in patients. Moreover, we discuss MRC-based cell therapy to prevent graft rejection and promote tolerance.

### Role of regulatory macrophages in organ transplantation

Historically, transplant immunologists have attempted to develop tolerogenic protocols by targeting the adaptive immune response. However, macrophage accumulation in the transplanted organ has long been recognized as a feature of allograft rejection. Inflammatory macrophages indeed initiate the allo-response against the transplanted organ [46] and represent a major cell subset during organ rejection (Fig. 2). Recent evidence suggests that macrophages are also important during the induction of transplantation tolerance [36]. The presence of graft-infiltrating macrophages with immunosuppressive activity has been described in long term surviving transplant recipients and is associated with unresponsiveness to the transplanted organ [47] (Fig. 2). In mice, inflammatory graft-infiltrating macrophages are Ly6C^high^ [48], while suppressive macrophages are Ly6C^low^ [49]. Inflammatory monocytes are rapidly recruited within the graft, where they can differentiate into either immunogenic Ly6C^high^ or tolerogenic Ly6C^low^ macrophages, depending on the local environment (Fig. 2). The behavior of migrating inflammatory monocytes observed in organ transplantation is similar to that reported in autoimmunity. In both conditions, the local microenvironment dictates the differentiation program of infiltrating monocytes: in inflamed tissues (organs early after transplantation or injured tissues) migrating monocytes acquire an inflammatory phenotype and differentiate into M1 or immunogenic DCs, that in turn promote T-cell activation and tissue damage. In steady-state conditions (engrafted transplanted organs or healthy tissues) migrating monocytes differentiate into anti-inflammatory/tolerogenic cells or M2 macrophages and contribute to tissue homeostasis and tolerance induction.

Therapeutic approaches preventing the accumulation of inflammatory monocytes/macrophages in the transplanted organs or improving their differentiation into suppressive cells have given promising results: selective depletion of inflammatory Ly6C^high^CCR2^high^ monocytes prolonged normoglycemia after allogeneic islet transplantation in streptozotocin-induced diabetic mice [50]. Alternatively, tolerogenic treatment with costimulatory blockade allowed inflammatory Ly6C^high^ monocytes infiltrating the allograft, early after transplantation, to differentiate into suppressive Ly6C^low^ macrophages through a CSF1-dependent mechanism [49]. These pre-clinical experiments show that targeting either donor- or recipient-derived monocytes represents a promising therapeutic approach to promote long-term graft acceptance in organ transplantation. However, there are no pharmacological agents that target monocyte/macrophages in clinical use. We believe that future experiments should consider the clinical development of immunotherapies that target myeloid cells within transplanted organs. One example is the potential use of drug-loaded nanoparticles [51]. The identification of new markers that allow specific targeting of myeloid cell subsets are required to facilitate the development of such innovative approaches.

### Contribution of dendritic cells in promoting/maintaining tolerance towards allo-antigens

As discussed above, tolDCs are essential for tolerance in autoimmunity, and recent evidence indicates that they also promote tolerance in the setting of transplantation (Fig. 2). TolDCs prevent pathogenic responses using a large arsenal of mechanisms: they promote T-cell anergy, clonal deletion, and apoptosis; they express and secrete immunomodulatory mediators that generate a pro-tolerogenic microenvironment that supports T-cell unresponsiveness and induction of Tregs (as reviewed in [52]) (Fig. 2). TolDCs generated with low doses of GM-CSF express heme-oxygenase 1 (HO-1), whose engagement prevents allogenic T-cell proliferation [53] and expression of Epstein-Barr virus-induced gene 3 (EBI3), a member of the IL-12 family [54]. Cardiac allograft survival induced by tolDC immunotherapy is abrogated by specific inhibition of HO-1 [55] or anti-EBI3 treatment [54]. These examples demonstrate that tolDCs generated with low doses of GM-CSF modulate T-cell responses and promote allograft tolerance via several mechanisms.

The transplantation procedure promotes a pro-inflammatory environment similar to that observed in the target organs of autoimmunity. In both instances, a hostile environment contributes to a reduction of the frequency of tolDCs and impairment of their immunosuppressive function. However, in contrast to most autoimmune settings, inflammation after organ transplantation is kept under control by the administration of immunosuppressive drugs that prevent immunogenic DC activation and consequently allo-reactive T-cell induction and function. Of note, these medications may also impair the induction and activity of tolDCs. Thus, the development of immunomodulatory agents that prevent inflammatory cell activation while favoring tolDC induction and function is a research priority in the setting of transplantation and autoimmune disease.

### Cell-based approaches to promote tolerance after allogeneic transplantation

The results obtained in experimental models of allo-transplantation and in proof-of-principle clinical trials in autoimmune diseases have prompted investigators to apply MRC-based cell approaches in the prevention of organ rejection. Hutchinson et al. developed a CSF1-dependent human suppressive myeloid cell-based medicinal product, called regulatory macrophages (Mregs) [56] and demonstrated the feasibility and safety of Mreg administration in a proof-of-principle clinical trial in living-donor renal transplant recipients [56]. A clinical-grade protocol for the manufacturing of Mregs has been optimized and donor-derived Mregs are currently pre-operatively administered to living-donor kidney transplant recipients under the umbrella of the ONE study (Clinicaltrials.gov: NCT02085629). The ONE study consortium (http://www.onestudy.org) is a European initiative of the FP7 7th Framework Programme ofthe European Union that aims at developing and comparing the safety and efficacy of various immunoregulatory cell products, including Mregs and tolDCs, as cell-based therapy in organ transplantation.

TolDCs suitable for cell-based therapy in transplantation can be generated by culturing precursors with several molecules including IL-10, TGF-β, VitD3, low dose of GM-CSF, rapamycin, tacrolimus or Dexa, or by downregulating costimulatory molecules (“Therapeutic intervention to restore tolerance in autoimmunity”). Regardless of the treatment, differentiated tolDCs express low levels of MHC-II and costimulatory molecules, are refractory to maturation, induce allogenic T-cell hypo-responsiveness in a mixed lymphocyte reaction, produce immunomodulatory mediators, and support Treg differentiation and proliferation [57].

The seminal study that led to the use of tolDCs as cell therapy in the field of transplantation stemmed from data demonstrating that adoptive transfer of donor-derived tolDCs prolonged heart graft survival in mice [58]. Since then, several studies in pre-clinical models of transplantation confirmed the immunosuppressive capacity of tolDCs, and, more recently, a meta-analysis showed the effectiveness of tolDCs in prolonging allograft survival [59]. Interestingly, donor-derived tolDCs, alone or in combination with immunosuppressive drugs, prolong cardiac allograft survival [52], but it was shown that recipient-derived tolDCs have a superior activity [60]. Adoptive transfer of tolDCs generated with VitD3 and IL-10 prolonged kidney allograft survival in a clinically relevant rhesus macaque model [61]. More recently, Cuturi et al. performed a proof-of-principle clinical trial with tolDCs generated in the presence of a low dose of GM-CSF as immunotherapy in kidney transplantation under the umbrella of the ONE study (Clinicaltrials.gov: NCT02252055).

The feasibility of generating ex vivo tolDCs for cell-based approaches has now been proven. However, the presence of inflammation generated by the transplant procedure and the use of immunosuppressive drugs may hinder the tolerogenic effects of tolDCs [52]. One way to counter this possibility is to inject semi-mature tolDCs, that are more resistant to this inflammatory environment and have been demonstrated to be effective in prolonging organ graft survival [52]. Furthermore, pre-clinical studies demonstrated that co-administration of immunosuppressive drugs in combination with tolDCs did not impair their activity (reviewed in [52]). The selection of the optimum immunosuppressive regimen that can sustain tolerance is an important consideration for the clinical application of tolDC-based therapy to prevent graft rejection.

## Conclusions and perspectives

Myeloid cells play a pivotal role in regulating innate and adaptive immune responses. They have a dual purpose, they can initiate effective controlled inflammation leading to activation of appropriate protective immune responses and they are involved in the resolution of inflammation and the promotion of tissue homeostasis and tolerance. Failure in either capacity has important consequences potentially leading to pathology. The discovery that several subtypes of myeloid cells with regulatory activity (MRCs) exist in vivo and can be induced in vitro opens the prospect of clinical interventions designed to induce/modulate these cells in vivo and use them as tolerogenic tool to re-establish/promote tolerance in autoimmune diseases and after transplantation. Recently, tolDC- and Mreg-based cell therapies have entered the clinical arena demonstrating the feasibility and safety of the approach. These encouraging results support the potential of using other subtypes of MRCs as a tolerogenic cell therapy in clinical practice.

A number of open questions remain regarding MRCs and their contribution to autoimmunity and transplantation: the most important is the identification of the relative contribution of each MRC subset in different pathological settings. The lack of consensus on markers to identify each MRC subset not only makes their identification in patients difficult, but also hampers the ability to specifically target the optimum cell type. In 2014, a European network (Mye-EUNITER, http://www.mye-euniter.eu/) was initiated under the umbrella of COST (European cooperation in science and technology) with the aim of joining forces to standardize the phenotypical and functional characterization of MRCs with the overall objective of expediting the application of this knowledge in diagnosis and treatment of a broad spectrum of disease. This is the first European initiative bringing together MRC experts from different research domains: basic, translational and clinical. By creating a forum for knowledge and expertise exchange, Mye-EUNITER will standardize tools to improve MRCs identification and characterization of their role in healthy and pathological conditions. This effort will contribute to increasing our knowledge about these particular subsets of myeloid cells and to identify strategies to target them at best or to use them as a cell-based product.

## Abbreviations

CNS: Central nervous system
Dexa: Dexamethasone
EAE: Experimental autoimmune encephalomyelitis
EBI3: Epstein-Barr virus-induced gene 3
HO-1: Heme-oxygenase 1
IBD: Inflammatory bowel disease
M-MDSCs: Monocytic myeloid-derived suppressor cells
M1: Pro-inflammatory macrophages
M2: Anti-inflammatory macrophages
MRCs: Myeloid regulatory cells
Mregs: Regulatory macrophages
MS: Multiple sclerosis
NETs: Neutrophil extracellular traps
pDCs: Plasmacytoid dendritic cells
PMN: Polymorphonuclear neutrophils
PMN-MDSCs: Granulocytic myeloid-derived suppressor cells
RA: Rheumatoid arthritis
SLE: Systemic lupus erythematosus
T1D: Type 1 Diabetes
tolDCs: Tolerogenic dendritic cells
VitD3: Vitamin D3
